# Design and overview of the Origins of Alzheimer’s Disease Across the Life course (ORACLE) study

**DOI:** 10.1007/s10654-020-00696-3

**Published:** 2020-12-16

**Authors:** Sander Lamballais, Maria C. Adank, Rowina F. Hussainali, Sarah Schalekamp-Timmermans, Meike W. Vernooij, Annemarie I. Luik, Eric A. P. Steegers, Mohammad Arfan Ikram

**Affiliations:** 1 Department of Epidemiology, Erasmus MC University Medical Center Rotterdam, PO Box 2040, 3000 CA Rotterdam, The Netherlands; 2 Generation R Study Group, Erasmus MC University Medical Center Rotterdam, Rotterdam, The Netherlands; 3 Department of Obstetrics and Gynecology, Erasmus MC University Medical Center Rotterdam, Rotterdam, The Netherlands; 4 Department of Radiology and Nuclear Medicine, Erasmus MC University Medical Center Rotterdam, Rotterdam, The Netherlands; 5 Department of Child and Adolescent Psychiatry/Psychology, Erasmus MC University Medical Center Rotterdam, Rotterdam, The Netherlands

**Keywords:** Brain, Cohort study, Epidemiology, Neuroimaging, Population-based, Risk factors

## Abstract

Brain development and deterioration across the lifespan are integral to the etiology of late-life neurodegenerative disease. Factors that influence the health of the adult brain remain to be elucidated and include risk factors, protective factors, and factors related to cognitive and brain reserve. To address this knowledge gap we designed a life-course study on brain health, which received funding through the EU ERC Programme under the name Origins of Alzheimer’s Disease Across the Life course (ORACLE) Study. The ORACLE Study is embedded within Generation R, a prospective population-based cohort study of children and their parents, and links this with the Rotterdam Study, a population-based study in middle-aged and elderly persons. The studies are based in Rotterdam, the Netherlands. Generation R focuses on child health from fetal life until adolescence with repeated in-person examinations, but has also included data collection on the children’s parents. The ORACLE Study aims to extend the parental data collection in nearly 2000 parents with extensive measures on brain health, including neuroimaging, cognitive testing and motor testing. Additionally, questionnaires on migraine, depressive symptoms, sleep, and neurological family history were completed. These data allow for the investigation of longitudinal influences on adult brain health as well as intergenerational designs involving children and parents. As a secondary focus, the sampling is enriched by mothers (n = 356) that suffered from hypertensive disorders during pregnancy in order to study brain health in this high-risk population. This article provides an overview of the rationale and the design of the ORACLE Study.

## Introduction

The number of Alzheimer’s disease (AD) cases is projected to double or even triple by 2050 [1, 2], which emphasizes the urgency to disentangle the etiology of AD and to develop effective preventative strategies. Although AD has an onset late in life, the risk to develop AD is influenced by both early-life and adulthood factors [3–5], including cognitive and academic performance [6–9], cardiovascular health [10–12], lifestyle factors [13–20] and life events [21–24]. These factors likely affect the susceptibility to develop AD through mechanisms such as cognitive and brain reserve [25, 26]. These mechanisms have been hypothesized to reduce or buffer the effect of brain pathology and aging.

The influence that the risk factors have on the incidence of AD likely depends on the life phase [27]. For example, hypertension during midlife has more influence on the risk of AD than hypertension later in life [28]. However, it is unclear whether hypertension during earlier phases of life also affect the incidence of dementia, and to what extent. Similarly, most studies on risk factors and compensatory mechanisms have primarily focused on midlife and beyond. It remains to be elucidated whether the risk factors already exert their effect on the etiology of AD during earlier phases of adulthood, and through which mechanisms.

Through the EU ERC Programme, funding was secured for a program entitled The Origins of Alzheimer’s Disease Across the Life course (ORACLE) Study, which aims to further elucidate the age at which AD risk factors start affecting brain health and to further understand the underlying mechanisms. The ORACLE Study is embedded within the Generation R Study [29], a prospective birth cohort established in 2002 that focuses on health development from fetal life until early adulthood. The parents of the children had a mean age of 30.9 years (standard deviation: 5.7) at study intake and participated in extensive measures of their health. The ORACLE Study started in 2017, as a dedicated research visit for the parents to conduct extensive measures on brain health, including neuroimaging and cognitive testing.

The ORACLE Study has several aims. The first aim is to elucidate the associations of established and promising AD risk factors that were collected during previous Generation R visits with cognitive and brain measures from the ORACLE research visit. Such factors will include vascular risk factors like blood pressure and lipid profiles, mental health metrics such as depressive-like symptoms, and lifestyle factors like daily exercise. A second aim is to consider how brain health develops over a lifetime. The ORACLE Study bridges the age gap between the children of the Generation R Study and participants from the Rotterdam Study [30], a prospective cohort study in individuals aged 45 years and older. By combining these three study populations, brain health can be studied across the life course. Furthermore, intergenerational effects can be examined by combining neuroimaging data from children and parents of the Generation R Study. A third aim is to study hypertensive disorders of pregnancy (HDP) and their impact on subsequent brain health and cognitive performance, as Generation R has extensive prenatal data. HDP affect 2–10% of all pregnancies and have been implicated as a potential risk factor for dementia [31]. However, little is known on how it influences subsequent brain health. The ORACLE Study will provide a unique opportunity to investigate the role of AD risk factors and HDP in brain health across adulthood.

In this manuscript, we give an overview of the measures that are collected from the parents as part of the ORACLE Study.

## Study overview

### Generation R study

**Fig. 1:**
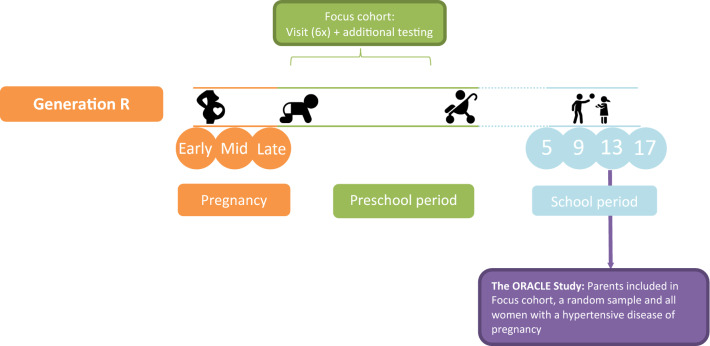
Flow of Generation R and the ORACLE Study. Parents were invited three times during pregnancy and returned to the research center with their children 5, 9 and 13 years after pregnancy. Additional detailed measurements of foetal and postnatal growth and development have been conducted in a subgroup of children (n = 1232, known as the ‘Focus cohort’) and their parents at 32 weeks gestational age and the postnatal ages of 1.5, 6, 14, 24, 36 and 48 months. The ORACLE Study started as part of the 13-year research visit

The ORACLE Study is embedded in the Generation R Study, a population-based prospective cohort study from fetal life onwards based in Rotterdam, the Netherlands [29, 32]. The Generation R Study was designed to identify early environmental and genetic causes of normal and abnormal growth, development and health from fetal life until young adulthood [29]. All pregnant women living in Rotterdam with an expected delivery date between April 2002 and January 2006 were invited to participate. A total of 9778 pregnant mothers and 6347 partners were recruited into the Generation R cohort, which led to 9749 live born children. A subset of 1232 children and their parents—the “Focus subcohort”—have partaken in additional detailed measurements of both fetal and postnatal growth and development. A schematic overview of the Generation R Study is shown in Fig. 1. All measures that have been performed in the parents of the Generation R Study have been described elsewhere [29, 32]. The Generation R Study and the ORACLE Study have been approved by Medical Ethical Committee of the Erasmus Medical Center Rotterdam, the Netherlands. All participants have to provide written informed consent before participating in the study.

### The ORACLE Study

The ORACLE Study is designed to test how factors during early adulthood affect brain health and structure at later ages. The parents within the Generation R Study provide a unique opportunity to tackle such questions. The parents had a mean age of 30.9 years (standard deviation: 5.7) at study baseline and data were collected for multiple factors that play a role in later adulthood brain health and the etiology of AD, like cardiovascular functioning, lifestyle and life events. To explore whether and how these factors measured during early adulthood affect consequent brain health in middle adulthood, we introduced the ORACLE Study. It consists of a parental research visit, with the goal to conduct cognitive testing, extensive neuroimaging and an assortment of other physiological and functional measures.

The ORACLE Study started in May 2017 and is still ongoing. The aim is to recruit 2,000 parents whose children had also participated in the most recent wave of the Generation R Study (2016–2019). We have invited all parents from the Focus subcohort. The sample is further supplemented by randomly selecting parents from the whole Generation R cohort until 2,000 individuals have participated.

The ORACLE Study aims to form a bridge between two population-based cohorts: The Generation R Study [29] and the Rotterdam Study [30]. The Generation R Study focuses on early life (childhood, adolescence), the ORACLE Study includes individuals during early and mid adulthood, and the Rotterdam Study covers mid adulthood until the end of life. By combining these three studies, brain health can roughly be studied from a life-course perspective. To harmonize the studies, the ORACLE Study has adapted the cognitive test battery from the Rotterdam Study as well as a similar set of brain magnetic resonance imaging (MRI) sequences.

### Hypertensive disorders of pregnancy

A secondary aim is to assess whether HDP affects the structure and function of the post-pregnancy brain. As such, we aim to invite all mothers who had experienced HDP during their index pregnancy. HDP was determined for every pregnancy during the initial phase of the Generation R Study. Obstetric records were obtained from the midwife and hospital registries [29, 33]. HDP was defined as pre-eclampsia and gestational hypertension. We used the criteria according to the International Society for the Study of Hypertension in Pregnancy of 2001 [34]. Therefore, gestational hypertension was defined as development of a systolic blood pressure ≥ 140 mmHg or a diastolic blood pressure ≥ 90 mmHg without proteinuria after 20 weeks of gestation in previous normotensive women. Preeclampsia was defined as a new onset of hypertension with a SBP ≥ 140 mmHg or a DBP ≥ 90 mmHg and proteinuria (≥ 300 mg/day) at or after 20 weeks of gestational age. A total of 356 women with HDP were eligible for inclusion, and they were invited irrespective of being part of the Focus subcohort.

## Measures

**Fig. 2:**
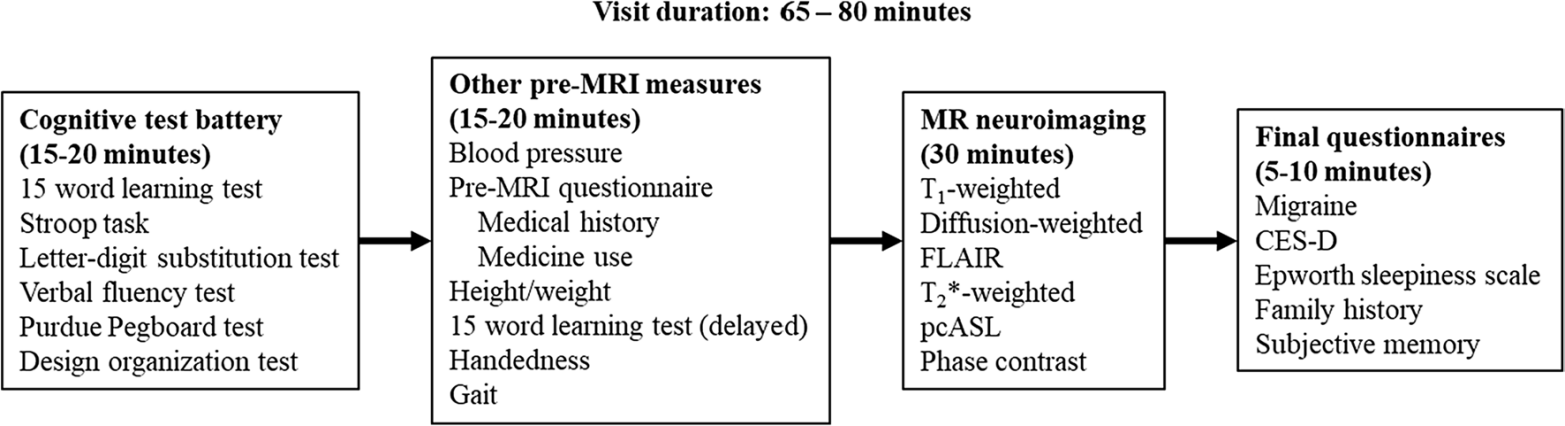
Schematic overview of the ORACLE Study research visit. *MRI* Magnetic resonance imaging, *FLAIR* Fluid-attenuated inversion recovery; *pcASL* Pseudo-continuous arterial spin labeling, *CES-D* Center for Epidemiological Studies – Depression scale

An overview of the ORACLE research visit and all measures is given in Fig. 2. The visit starts with a cognitive test battery consisting of six tests (see “Cognitive testing” section): (1)the 15-word learning test, (2) the Stroop task, (3) a letter-digit substitution test, (4) a verbal fluency test, (5) the Purdue pegboard test and (6) the design organization test. The cognitive battery is followed by an assessment of gait (“Gait assessment” section), a blood pressure measurement (“Blood pressure and anthropometry” section) and questionnaires (“Questionnaires” section). The participants are then scanned in an MRI scanner, and the session lasts for 30 min (“Neuroimaging” section). The total visit duration is approximately 65 to 80 min.

### Neuroimaging

The aims of the neuroimaging are to:

- map and quantify the structure of the gray and white matter of the brain;
- map and quantify markers of cerebrovascular disease, i.e. white matter hyperintensities, brain infarcts (lacunar and cortical) and microbleeds;
- map and quantify the cerebral blood flow.

We chose sequences and acquisition parameters that were comparable to the acquisition protocols of the Generation R Study and the Rotterdam Study while constraining the scanning time to 30 min. The finalized scan protocol is shown in Table 1.

**Table 1:** Parameters for the MRI sequences within the ORACLE Study

Sequence	Comment	Mode	Time (min:s)	TR/TE (ms)	TI (ms)	Flip angle (°)	FOV (cm^2^)	Matrix	Number of slices	Slice thickness (mm)	BW (kHz)
IR-FSPGR	ARC acceleration = 2	3D	4:48	8.8/3.4		10	22	220 × 220	196	1	25
DWI	35 directions; b = 1000 mm^2^/s, b_0_ = 3; PED P/A	3D	5:59	8200/84			24	120 × 120	65	2	250
DWI	b_0_ = 3; PED A/P	3D	0:39	8200/84			24	120 × 120	65	2	250
FLAIR		2D	4:42	8761/120	2281		25	320 × 224	64	2.5	50
T2*	GRE	3D	5:54	min/23.9		12	25	320 × 224	160	1	41.67
pcASL	3 PLDs: 1000, 1570 and 2460 ms	3D	3:16	5591/10.7			24		36	4	62.50
PC	Carotid and basilar flow; VENC = 80 cm/s	3D	1:53	min/3.8		10	23	256 × 160	20	2	41.67

*TR* Repetition time, *TE* Echo time, *TI* Inversion time, *FOV* Field of view, *BW* Bandwidth, *IR-FSPGR* Inversion recovery fast spoiled gradient recalled, *DWI* diffusion weighted imaging, *FLAIR* Fluid-attenuated inversion recovery, *pcASL* Pseudo-continuous arterial spin labeling, *PC* Phase contrast, *ARC* Autocalibrating reconstruction for Cartesian imaging, *PED* Phase-encoding direction, *P/A* Posterior-anterior, *A/P* Anterior–posterior, *GRE* Gradient echo, *PLD* Postlabeling delay, *VENC* Velocity encoding

Participants are excluded if they have any contraindications for the MRI, like metal implants or claustrophobia.

#### Scanner and equipment

As of 2013, the Generation R Study has a dedicated MRI scanner in the Erasmus MC-Sophia hospital [35], the same hospital that houses the Generation R research center. We are performing the ORACLE Study on this MRI scanner as well, in order to make the images more comparable to the images of the Generation R Study children. The scanner is a 3 T GE Discovery MR750w MRI System (General Electric, Milwaukee, WI, USA) with the GE DV24 software package. The software package has intentionally not been updated since 2014, to ensure that the images remain relatively unchanged over the years. Images are obtained using an eight-channel head coil.

Several steps are undertaken to ensure comfort of the participants and to reduce participant motion. To reduce noise levels the participants are given earplugs and additionally headphones if this fits into the head coil. To ensure immobility of the head we use bilateral soft cushioning. A stiff cushion is also placed under the legs of the participants to reduce discomfort during scanning. Participants with back problems are offered additional pillows and other support. Finally, all participants are shown the same nature documentary during the scanning unless they prefer not to.

#### Image acquisition

The session starts with a three-plane localizer for positioning, and an ASSET scan to enable parallel imaging. T_1_-weighted images to assess the structure of the brain are obtained using a 3D axial inversion recovery fast spoiled gradient recalled sequence (1 × 1 × 1 mm^3^). The sequence is further accelerated by a factor of 2 using autocalibrating reconstruction for Cartesian imaging (ARC). White matter microstructural integrity is assessed with an axial spin echo sequence with an echo planar imaging (EPI) readout (2 × 2 × 2 mm^3^). The gradient is set at b = 1000 m/s^2^ in 35 directions with a posterior-anterior phase encoding direction. In addition, 3 sets of images with a gradient of b = 0 m/s^2^ are collected. To be able to perform susceptibility distortion correction we also collect 3 sets of b = 0 m/s^2^ images with an anterior–posterior phase encoding direction.

White matter lesions and infarcts are visualized using a 2D axial fluid-attenuated inversion recovery (FLAIR) sequence (0.8 × 1.1 × 2.5 mm^3^). To visualize microbleeds we use a T_2_*-weighted sequence (0.8 × 1.1 × 1.0 mm^3^). Both of these are based on the sequence parameters of the Rotterdam Study to promote cross-study comparisons [36]. We further assess local blood perfusion with a pseudo-continuous arterial spin labeling (pcASL) sequence with three postlabeling delays (1000, 1570 and 2460 ms). The total cerebral blood flow is quantified using an ungated 3D phase contrast sequence (velocity encoding = 80 cm/s) and based on the blood flow through the carotids and the basilar arteries.

**Fig. 3:**
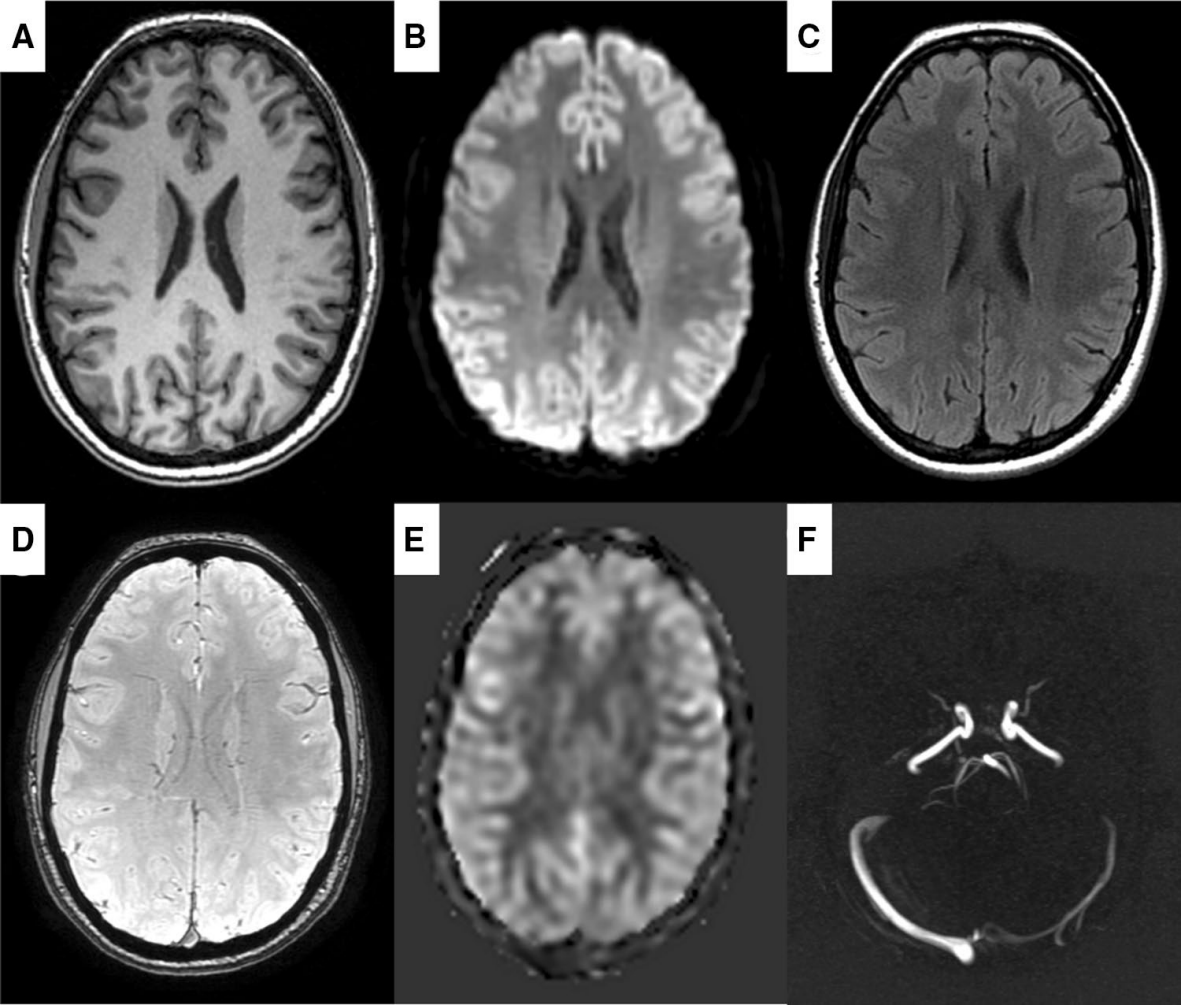
Example images from all sequences in the MRI protocol. **a** T_1_-weighted, **b** diffusion-weighted, **c** FLAIR, **d** T_2_*-weighted, **e** pcASL, **f** phase contrast

Examples of each sequence have been compiled into Fig. 3. Once the scanning session is completed, the images are send and stored in an XNAT storage instance [37].

#### Quality assessment

An initial assessment of general image quality is made by the radiographer during the scanning for the T_1_-weighted sequence. The quality of the T_1_-weighted scans is classified as poor, questionable, good or excellent. In most cases, poor quality scans seem to be due to excessive movement. If the scans are rated as poor or questionable, the radiographer instructs the participant to try to lay as still as possible, and that the T_1_-weighted sequence will be repeated.

Image quality of all sequences is further assessed during and after image processing with manual and automated methods. T_1_-weighted segmentations are visually inspected by trained raters and each scan receives a fail/pass rating on a global level. This is done by rating a subset of slices in all three orientations (i.e. axial, coronal and sagittal) as well as the 3D reconstructions of the white and pial surfaces.

T_1_-weighted images are also processed through a validated and automated quality assessment pipeline [38]. Images with low ratings from this pipeline are reinspected by the raters, and excluded if justified. Furthermore, the quality rating from the automated pipeline will be used as a covariate in MRI-related projects, to see whether the analyses are confounded by subtle differences in image and processing quality.

#### Incidental findings

A by-product of population-based neuroimaging is the detection of incidental findings (IFs), i.e. abnormalities that are unrelated to the aims of the study but could bear clinical relevance. A meta-analysis study estimated the prevalence of such findings for brain MRI to be 2.7%, with neoplastic findings in about 0.7% of individuals [39]. However, it remains unclear what the best course of action is for incidental findings. For example, a follow-up study found that small meningiomas, the most common neoplastic finding, tend to remain stable and thus clinical intervention is not needed [40]. The UK Biobank also found that the majority of clinical referrals of incidental findings did not benefit the participants while being accompanied by side effects such as emotional distress [41]. Incidental findings are thus to be expected but should be approached conservatively, to minimize burden on the participants.

Similar to the Generation R child neuroimaging waves we opted for a two-layered approach in incidental finding detection and management [35]. The first layer takes place during the scanning session itself. The radiographer scrolls through the T_1_-weighted images once, to detect any gross abnormalities. If any are detected, a certified neuroradiologist is contacted (author: MWV). The second layer is post-hoc inspection of the T_1_-weighted images, the DWI images, the FLAIR images and the T2*-weighted images by trained personnel. A certified neuroradiologist subsequently checks all findings (author: MWV). When a finding is deemed clinically relevant, it is discussed in a broader consensus meeting where the decision is made to refer the patient for further diagnostic testing and/or follow-up or clinical intervention.

### Cognitive testing

All participants are tested individually by trained examiners. The participants are told that they will take part in several tests and are asked to try their best. If participants ask about the purpose of the test, the experimenter indicates that they cannot disclose this during the test battery. If participants indicate that they want to restart or that they feel like they are doing poorly, the experimenter encourages them to continue and finish the test. Spare glasses are available for visually impaired participants who did not bring their own. Audio is recorded for the entire cognitive test battery if the participant consented. If needed, a stopwatch is used to measure time.

#### 15-word learning test

The 15-word learning test is a neuropsychological test to assess the ability of verbal learning, retrieval and recognition of verbal memory [42]. The test consists of three subtasks [43]. The first subtask consists of three trials where the same 15 unrelated words were used as stimuli. During each trial, the words are shown one by one on a computer screen, with a presentation time of 2 s per word, in the same order. After every trial, the participant is asked to name as many words as they can remember (immediate recall), and the trial is ended once the participant cannot recall any more words.

At least 20 min after the third trial, the participant is asked to name as many presented words as they can remember (delayed recall). Once the participant named all words that they can recall, they are presented with 15 previously shown words and 15 new words on the computer screen, one by one. The participants are asked to answer ‘Yes’ or ‘No’ to whether the item belonged in the list of immediate recall (recognition). The number of correctly recalled words in each trial are scored as the main outcome measures [42].

#### Stroop task

The Stroop task is a neuropsychological test used to assess the ability to inhibit cognitive interference. Cognitive interference occurs when the processing of a specific stimulus feature impedes the simultaneous processing of a second stimulus attribute, known as the Stroop Effect [44, 45]. The Stroop task that is being used consists of three subtasks, and 40 stimuli for each subtask are distributed evenly in a 4 by 10 matrix [46]. The first subtask shows words of different colors (red, yellow, blue, or green) in black writing. The second subtask shows rectangles solidly colored in either red, yellow, blue or green. The third and last task shows the words of different colors (red, yellow, blue, or green) in dissimilar ink color [46]. Every task must be read aloud as quickly as possible without mistakes. There is no time limit to complete each subtask. The time in seconds needed for a subtask is given as dependent measures (reading subtask, color naming subtask, and color-word interference subtask respectively) [42].

#### Letter-digit substitution test

The Letter-digit substitution test is a neuropsychological test to assess the ability of processing speed and executive function [42]. In the test, a key in which the numbers 1 to 9 are paired with a letter is given at the top of the worksheet [47]. Beneath the key, letters are given in a random order and the participant needs to pair the letters with the number according to the key. The first ten items are used for practice to ensure that the participant understands the test instructions. Following the practice round, the participant needs to pair as many numbers with the letters within 60 s in the given order. The number of correct items is used as the outcome of the test, with the maximum being 125 points [42].

#### Verbal fluency test

The verbal fluency test is a neuropsychological test to assess the ability of the efficiency of searching in long-term memory [42]. An animal-based task is used, where participants are asked to generate as many animal names as possible within 60 s [48]. The number of correct animals is used as the outcome of the test [42]. Furthermore, the examiners write out the answers in full for post-hoc construction of semantic networks across all participants [49, 50]. This can be done to determine the degree to which participants cluster within and switch between semantic categories of animals [50].

#### Purdue pegboard test

The Purdue pegboard test is a neuropsychological test to assess dexterity and fine motor skill [42]. The Purdue pegboard is a rectangular board consisting of two columns of 25 holes [51, 52]. Above these columns are reservoirs for metal pins. The participant needs to move the pins one at a time into the holes of the column on the same side of the board as the hand being used. They start at the top of the column and place as many pins as possible within 30 s. The task consists of three subtasks [51, 52]. The first task is with the dominant hand, followed by the non-dominant hand, and then using the left and right hand simultaneously. The numbers of moved pins for each subtask are used as the outcome measure [42].

#### Design organization test

The design organization test is a neuropsychological test to assess visuospatial ability [42, 53]. The participant is presented with nine designs consisting of squares. Six different squares are used: 1 black square, 1 white square, and 4 squares that are half-black and half-white divided along the diagonal in different orientations. Numbers from 1 to 6 are assigned to each type of square, with a key at the top of the sheet. Participants have to convert the pattern designs to corresponding number designs. They have to fill in as many numbers as possible within 120 s. The first nine patterns consist of 5 designs with 2 by 2 squares, followed by 4 designs with 3 by 3 squares, and thus a maximum of 56 points can be scored. The number of correct items completed is used as the outcome measure [42].

### Gait assessment

The walking pattern, or gait, is a complex sequence of movements integrating sensory information and motor commands [54–56]. Gait is considered an accurate reflection of general health and is influenced by many organ systems such as the central and peripheral nervous system, cardiovascular system, and musculoskeletal system [57–59]. Gait is assessed with an electronic walkway using pressure sensors (GAITRite; Sparta, NJ: 4.88-m active area; 120-Hz sampling rate) and is considered accurate to determine gait parameters. Participants perform a standardized gait protocol consisting of four different walking conditions: normal walk, turning, tandem walk, and dual task. In the normal walk, participants walk six times over the walkway at their own pace. In turning, participants walk at their usual pace, turn halfway and return to the starting position. In the tandem walk, participants walk tandem (heel-to-toe) on the walkway. During the dual task, participants have to count down from a 100 by seven while walking over the walkway. Raters inspect all recordings and identify individual footsteps. The GAITRite software returns a broad range of parameters that are commonly summarized into seven independent gait domain: rhythm, phases, variability, pace, tandem, turning, and base of support. Until February 2018, the gait data were collected with the wireless GAITRite CIRFace system. We experienced intermittent technical issues primarily related to loss of wireless signal. Since March 2018, the gait data has been collected with the wired GAITRite RE system. Both systems have the same settings and characteristics, and so the data from both systems will be used for analysis.

### Blood pressure and anthropometry

Systolic and diastolic blood pressure are measured with the validated automatic sphygmomanometer Omron 907 (OMRON, Matsusaka Co., Ltd., Japan) [60]. All participants are seated in upright position with back support. Blood pressure is measured two times over a 60-s interval, and the mean blood pressures are used for further analysis [61]. Furthermore, body weight (kilograms) and body height (centimeters) are measured after participants take off their footwear.

### Questionnaires

#### Migraine

Migraine is assessed with a validated screening questionnaire [62]. This questionnaire includes five questions asking whether the participant had (i) severe headaches in the past 12 months, (ii) what the headache severity was, (iii) whether the participant had suffered from headaches which were preceded by visual disturbances, (iv) whether the participant had been diagnosed with migraine by a physician, and (v) whether the participant had ever used anti-migraine medication.

#### Depressive symptoms

Depressive symptoms are measured using the validated Dutch version of the Center for Epidemiologic Studies-Depression Scale (CES-D) [63]. The CES-D comprises 20 items, each with a possible score of 0–3, and the score ranges from 0–60.

#### Sleep propensity

Sleep propensity, one’s readiness to transition from an awake state to sleep, is measured with a Dutch version of The Epworth Sleepiness Scale [64]. The scale comprises 8 items, each with a possible score of 0–3, and the score ranges from 0 to 24. Higher scores indicate higher levels of sleep propensity.

#### Handedness

Handedness is assessed using a modified version of the Edinburgh Handedness Inventory [65]. This inventory contains questions on which hand someone prefers for a range of activities such as writing, holding a fork and striking a match. An option was added for both hands, to capture ambidexterity. Furthermore, an item on eyedness (“which eye would you look with through a telescope”) and footedness (“which leg would you use to kick a ball”) were included as well. From these items, a laterality index ranging from -1 (preference for left) to 1 (preference for right) is calculated.

#### Subjective memory complaints

Subjective memory complaints are assessed with four self-reported question. The first question is “do you have more difficulty remembering things?” If participants answer “Yes”, three follow-up questions are asked: the year in which these problems seemed to start, whether the problems started suddenly (no/yes), and whether the problems have changed over time (no/yes).

#### Other self-reported data

Additional information is obtained from participants through a semi-structured interview and a separate questionnaire. Participants are asked about their sleep during the last night (shorter, longer or the same as usual), and consumption of caffeine, nicotine, alcohol and drugs in the past 24 h. Furthermore, participants are asked about their medical status (presence of diseases) as well as medication use. We specifically ask about neurological and psychiatric diseases and disorders. Women are asked about their menstrual cycle, i.e. whether they menstruate and when they last menstruated. Finally, participants are asked whether their biological mother or biological father have been diagnosed with dementia, Parkinson’s disease, schizophrenia or bipolar disease.

## Current state of recruitment

Below we present the progress of the ORACLE Study from May 2017 up to September 2019. In total, 1579 individuals have been reached for an invitation to take part in the ORACLE Study, and 1307 (83%) have accepted that invitation. Of the respondents, 169 were HDP women, and 135 (80%) participated. Most participants were female (n = 842, 64.4%). The mean age during the visit was 46.4 years for the women (standard deviation: 4.4, range: 33.0–60.9) and 49.0 for the men (standard deviation: 5.0, range: 36.5–72.0). The mean follow-up time since the intake of the Generation R Study was 14.9 years (standard deviation: 0.8 years). Most participants (80.6%) reported to be of Dutch ancestry, 15.0% as non-Western and 4.4% as other Western. Finally, at study intake 58.8% of the participants had a university degree, 37.8% only finished secondary (vocational) education, and 3.4% only finished primary school or had no degree. An overview of the participation rates for each measurement is given in Table 2. Out of 1307 participants, 1280 (97.9%) completed at least one MRI sequence. The remaining participants did not participate in neuroimaging due to claustrophobia (n = 11), contraindications (n = 9), technical scan issues (n = 5) and participants having to leave early (n = 2). Of the scanned individuals, 1180 (92.2%) had complete data on all sequences (99.8% T_1_-weighted, 98.8% diffusion-weighted imaging, 99.1% FLAIR, 97.4% T_2_*-weighted, 95.2% pcASL and 96.4% phase contrast). Early termination of a scan session was most commonly due to anxious feelings. Furthermore, the pcASL and the phase contrast were sometimes skipped due to time constraints if the T_1_-weighted sequence had to be rescanned for quality purposes. Finally, the diffusion-weighted sequence was not performed in the first 11 participants due to technical issues.

**Table 2:** Participation rates for each measurement of the ORACLE Study up to September 2019

Measurement	Eligible (n)	Available (n)	Participation (%)
*MRI*			
T_1_-weighted	1280	1277	99.8
Diffusion-weighted imaging	1269	1254	98.8
FLAIR	1280	1269	99.1
T_2_^*^-weighted	1280	1247	97.4
pcASL	1280	1219	95.2
Phase contrast	1280	1234	96.4
*Cognitive testing*			
15-word learning test	1307	1300	99.5
Stroop task	1307	1300	99.5
Letter-digit substitution test	1307	1300	99.5
Verbal fluency test	1307	1300	99.5
Purdue pegboard test	840^a^	820	97.6
Design organization test	839^a^	830	98.9
*Other measures*			
Gait assessment	1307	1016	78.0
Blood pressure	905^a^	889	98.2
Anthropometry (weight, height)	1307	1302	99.6
Migraine questionnaire	905^a^	872	96.4
CES-D	905^a^	885	97.8
ESS	905^a^	884	97.7
Handedness	1307	1302	99.6
Subjective memory complaints	905^a^	879	97.1

^a^These measures were introduced later in the study

Participation rates for the other tests were generally close to 100% (Table 2). A number of measures have lower participation rates as they were introduced at a later time point during data collection, i.e. the Purdue pegboard test, the design organization test, the blood pressure measurements and some of the questionnaires. In addition, the gait assessment experienced technical issues during the initial phase of the data collection, which resulted in a relatively low participation rate (78.0%). Other non-participation can be explained by participant refusal or inability to participate (e.g. muscle problems for the Purdue pegboard test), technical problems with the equipment (e.g. blood pressure measurements), or skipping of questions in questionnaires.

## Discussion

Given the high response rates and the high quality of the collected data, the ORACLE Study will likely reach its goal of 2000 participants with a few more months of data collection. Once the data collection is completed the ORACLE Study will be open for collaborative projects. All requests for collaboration can be directed to study PI Professor M. Arfan Ikram (m.a.ikram@erasmusmc.nl).
